# The genome sequence of the Yellow Horned moth, *Achlya flavicornis *(Linnaeus, 1758)

**DOI:** 10.12688/wellcomeopenres.19846.2

**Published:** 2025-02-19

**Authors:** Liam M. Crowley, Dominic Phillips, University of Oxford and Wytham Woods Genome Acquisition Lab, Darwin Tree of Life Barcoding collective, Wellcome Sanger Institute Tree of Life programme, Wellcome Sanger Institute Scientific Operations: DNA Pipelines collective, Tree of Life Core Informatics collective, Darwin Tree of Life Consortium

**Affiliations:** 1 University of Oxford, Oxford, England, UK; 2 Natural History Museum, London, England, UK

**Keywords:** Achlya flavicornis, Yellow Horned, genome sequence, chromosomal, Lepidoptera

## Abstract

We present a genome assembly from an individual male *Achlya flavicornis* (the Yellow Horned; Arthropoda; Insecta; Lepidoptera; Drepanidae). The genome sequence is 444.6 megabases in span. Most of the assembly is scaffolded into 30 chromosomal pseudomolecules, including the Z sex chromosome. The mitochondrial genome has also been assembled and is 15.34 kilobases in length. Gene annotation of this assembly on Ensembl identified 17,588 protein coding genes.

## Species taxonomy

Eukaryota; Metazoa; Eumetazoa; Bilateria; Protostomia; Ecdysozoa; Panarthropoda; Arthropoda; Mandibulata; Pancrustacea; Hexapoda; Insecta; Dicondylia; Pterygota; Neoptera; Endopterygota; Amphiesmenoptera; Lepidoptera; Glossata; Neolepidoptera; Heteroneura; Ditrysia; Obtectomera; Drepanoidea; Drepanidae; Thyatirinae; *Achlya*; *Achlya flavicornis* (Linnaeus, 1758) (NCBI:txid753485).

## Background

The Yellow Horned, *Achlya flavicornis* is a medium-sized Drepanid moth, with a forewing length of 17 to 20 mm. It is characterised by a grey speckled appearance, with a distinctive white spot near the centre leading edge of the wing, and dark lines crossing the forewing. Both common and scientific names of *A. flavicornis* are derived from its orangish antennae coupled with its yellowish tufted scape ( British Lepidoptera, 2022). An early-flying monovoltine species, *A. flavicornis* flies between late February and mid-April in the UK. It overwinters under leaf litter in a thin cocoon as a pupa ( Waring *et al.*, 2017).

*A. flavicornis* is found in woodland, heathland and moorland habitats with birch scrub; the larva feeds on silver birch ( *Betula pendula*) and downy birch ( *Betula pubescens*) ( Waring *et al.*, 2017).

*Achlya flavicornis* is generally widespread through the western Palaearctic, from northern Spain to Norway, crossing down into central Russia, but there are relatively few records from eastern Russia and its range extends eastwards to the northern Japan, with sparse records there ( GBIF Secretariat, 2022). Populations in the UK appear to have increased since 1970 ( Conrad *et al.*, 2006); this could be due to its ability to thrive in open nutrient-poor habitat types ( Tyler, 2020). It is a common resident species in Britain and Ireland, with two subspecies: ssp. *galbanus* being well distributed on clays and light calcareous soils in England and lowland Wales, and ssp. *scotia* having a wide distribution in mainland Scotland, the Orkney Isles and the Inner Hebrides ( Waring *et al.*, 2017).

The full genome for *Achlya flavicornis* may provide insight into how it is able to thrive in habitats which prove too harsh for other closely related moth species. The genome of the Yellow Horned was sequenced as part of the Darwin Tree of Life Project, a collaborative effort to sequence all named eukaryotic species in the Atlantic Archipelago of Britain and Ireland. Here we present a chromosomally complete genome sequence for *A. flavicornis*, based on one male specimen.

## Genome sequence report

**Figure 1.**
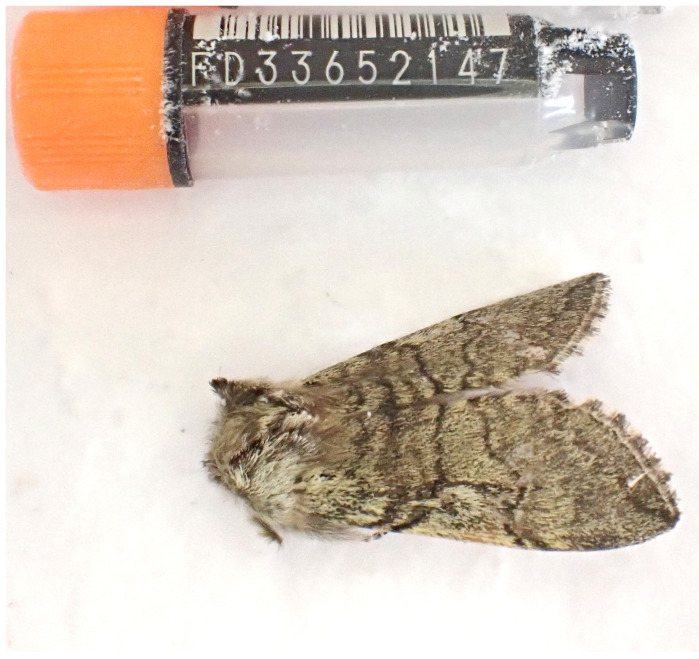
Photograph of the *Achlya flavicornis* (ilAchFlav2) specimen used for genome sequencing.

The genome was sequenced from one male *Achlya flavicornis* ( Figure 1) collected from Wytham Woods, Oxfordshire, UK (51.77, –1.34). A total of 49-fold coverage in Pacific Biosciences single-molecule HiFi long reads was generated. Primary assembly contigs were scaffolded with chromosome conformation Hi-C data. Manual assembly curation corrected five missing joins or mis-joins and removed one haplotypic duplication, reducing the scaffold number by one.

**Figure 2.**
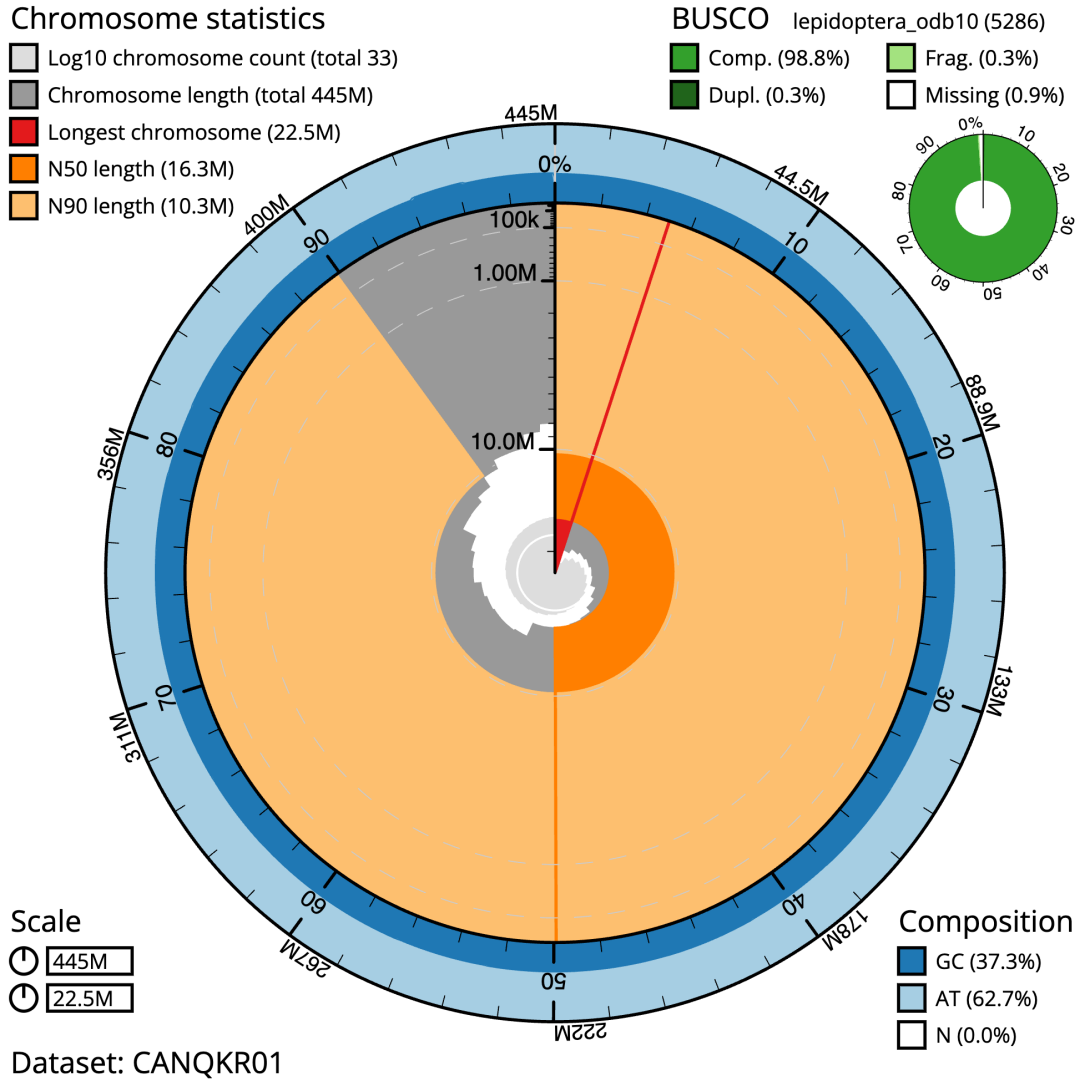
Genome assembly of *Achlya flavicornis*, ilAchFlav2.1: metrics. The BlobToolKit Snailplot shows N50 metrics and BUSCO gene completeness. The main plot is divided into 1,000 size-ordered bins around the circumference with each bin representing 0.1% of the 444,573,508 bp assembly. The distribution of sequence lengths is shown in dark grey with the plot radius scaled to the longest sequence present in the assembly (22,518,559 bp, shown in red). Orange and pale-orange arcs show the N50 and N90 sequence lengths (16,269,518 and 10,316,657 bp), respectively. The pale grey spiral shows the cumulative sequence count on a log scale with white scale lines showing successive orders of magnitude. The blue and pale-blue area around the outside of the plot shows the distribution of GC, AT and N percentages in the same bins as the inner plot. A summary of complete, fragmented, duplicated and missing BUSCO genes in the lepidoptera_odb10 set is shown in the top right. An interactive version of this figure is available at https://blobtoolkit.genomehubs.org/view/ilAchFlav2.1/dataset/CANQKR01/snail.

The final assembly has a total length of 444.6 Mb in 32 sequence scaffolds with a scaffold N50 of 16.3 Mb ( Table 1). Most (99.98%) of the assembly sequence was assigned to 30 chromosomal-level scaffolds, representing 29 autosomes and the Z sex chromosome. Chromosome-scale scaffolds confirmed by the Hi-C data are named in order of size. The Z chromosome was identified based on synteny with *Habrosyne pyritoides* (GCA_907165245.1; ilHabPyri1). ( Figure 2– Figure 5; Table 2). While not fully phased, the assembly deposited is of one haplotype. Contigs corresponding to the second haplotype have also been deposited. The mitochondrial genome was also assembled and can be found as a contig within the multifasta file of the genome submission.

**Table 1.** Genome data for *Achlya flavicornis*, ilAchFlav2.1.

Project accession data		
Assembly identifier	ilAchFlav2.1	
Species	*Achlya flavicornis*	
Specimen	ilAchFlav2	
NCBI taxonomy ID	753485	
BioProject	PRJEB56908	
BioSample ID	SAMEA110451568	
Isolate information	ilAchFlav2, male: head and thorax (DNA sequencing and Hi-C scaffolding)	
Assembly metrics *		*Benchmark*
Consensus quality (QV)	65.6	*≥ 40*
*k*-mer completeness	100%	*≥ 95%*
BUSCO **	C:98.8%[S:98.5%,D:0.3%], F:0.3%,M:0.9%,n:5,286	*C ≥ 95%*
Percentage of assembly mapped to chromosomes	99.98%	*≥ 90%*
Sex chromosomes	Z chromosome	*localised homologous pairs*
Organelles	Mitochondrial genome assembled	*complete single alleles*
Raw data accessions		
PacificBiosciences SEQUEL II	ERR10439744	
Hi-C Illumina	ERR10395994	
Genome assembly		
Assembly accession	GCA_947623365.1	
*Accession of alternate haplotype*	GCA_947623295.1	
Span (Mb)	444.6	
Number of contigs	63	
Contig N50 length (Mb)	11.8	
Number of scaffolds	32	
Scaffold N50 length (Mb)	16.3	
Longest scaffold (Mb)	122.5	
Genome annotation		
Number of protein-coding genes	17,588	
Number of gene transcripts	17,770	

* Assembly metric benchmarks are adapted from column VGP-2020 of “Table 1: Proposed standards and metrics for defining genome assembly quality” from ( Rhie *et al.*, 2021). ** BUSCO scores based on the lepidoptera_odb10 BUSCO set using v5.3.2. C = complete [S = single copy, D = duplicated], F = fragmented, M = missing, n = number of orthologues in comparison. A full set of BUSCO scores is available at https://blobtoolkit.genomehubs.org/view/ilAchFlav2.1/dataset/CANQKR01/busco.

**Figure 3.**
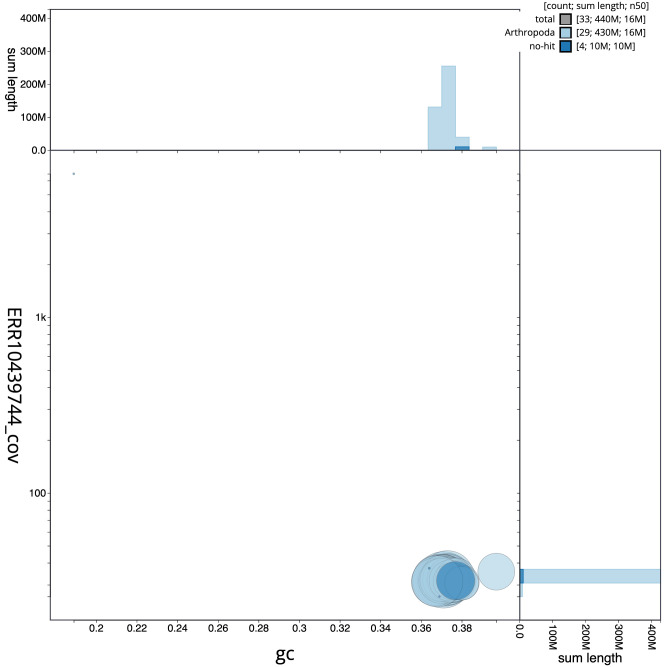
Genome assembly of *Achlya flavicornis*, ilAchFlav2.1: BlobToolKit GC-coverage plot. Scaffolds are coloured by phylum. Circles are sized in proportion to scaffold length. Histograms show the distribution of scaffold length sum along each axis. An interactive version of this figure is available at https://blobtoolkit.genomehubs.org/view/ilAchFlav2.1/dataset/CANQKR01/blob.

**Figure 4.**
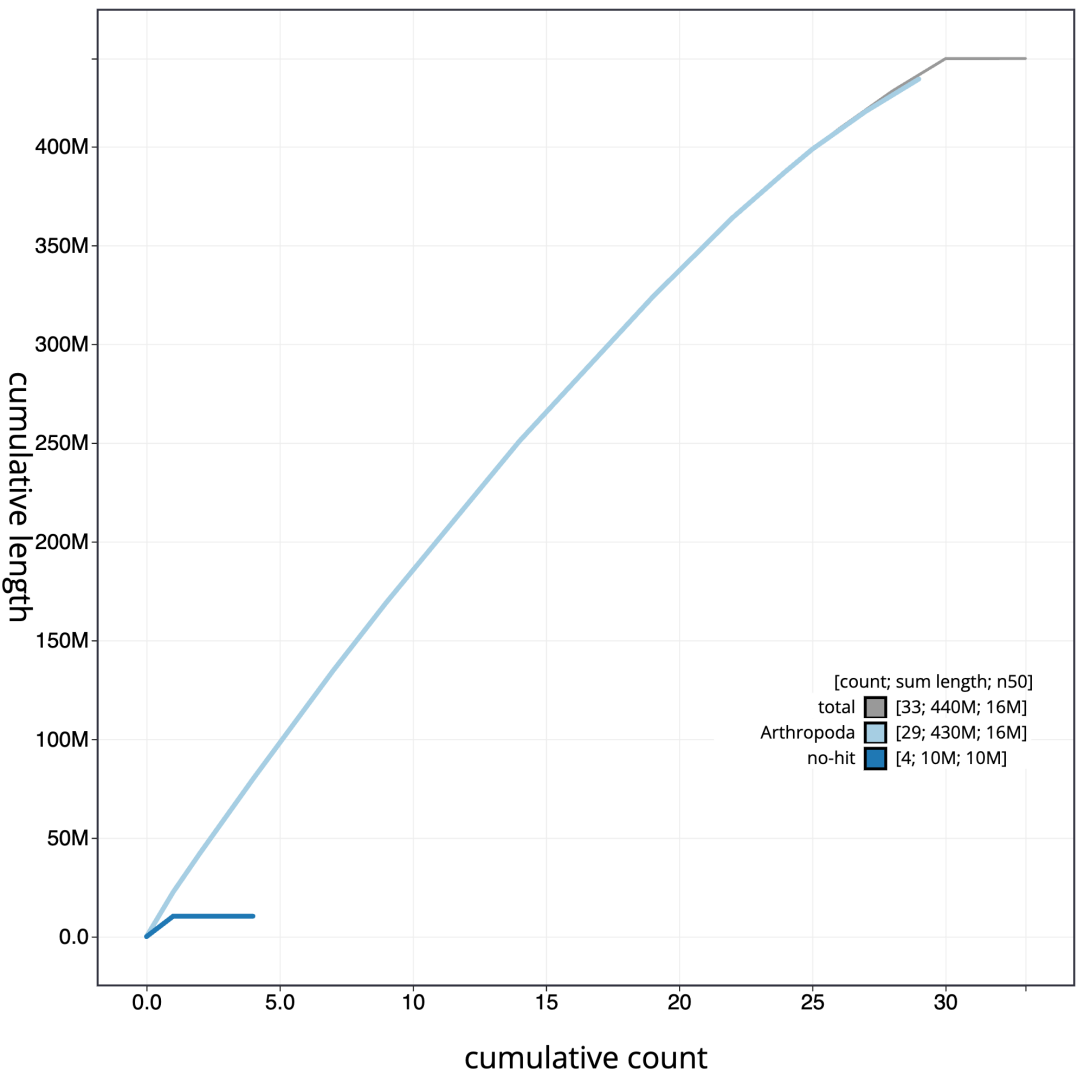
Genome assembly of *Achlya flavicornis*, ilAchFlav2.1: BlobToolKit cumulative sequence plot. The grey line shows cumulative length for all scaffolds. Coloured lines show cumulative lengths of scaffolds assigned to each phylum using the buscogenes taxrule. An interactive version of this figure is available at https://blobtoolkit.genomehubs.org/view/ilAchFlav2.1/dataset/CANQKR01/cumulative.

**Figure 5.**
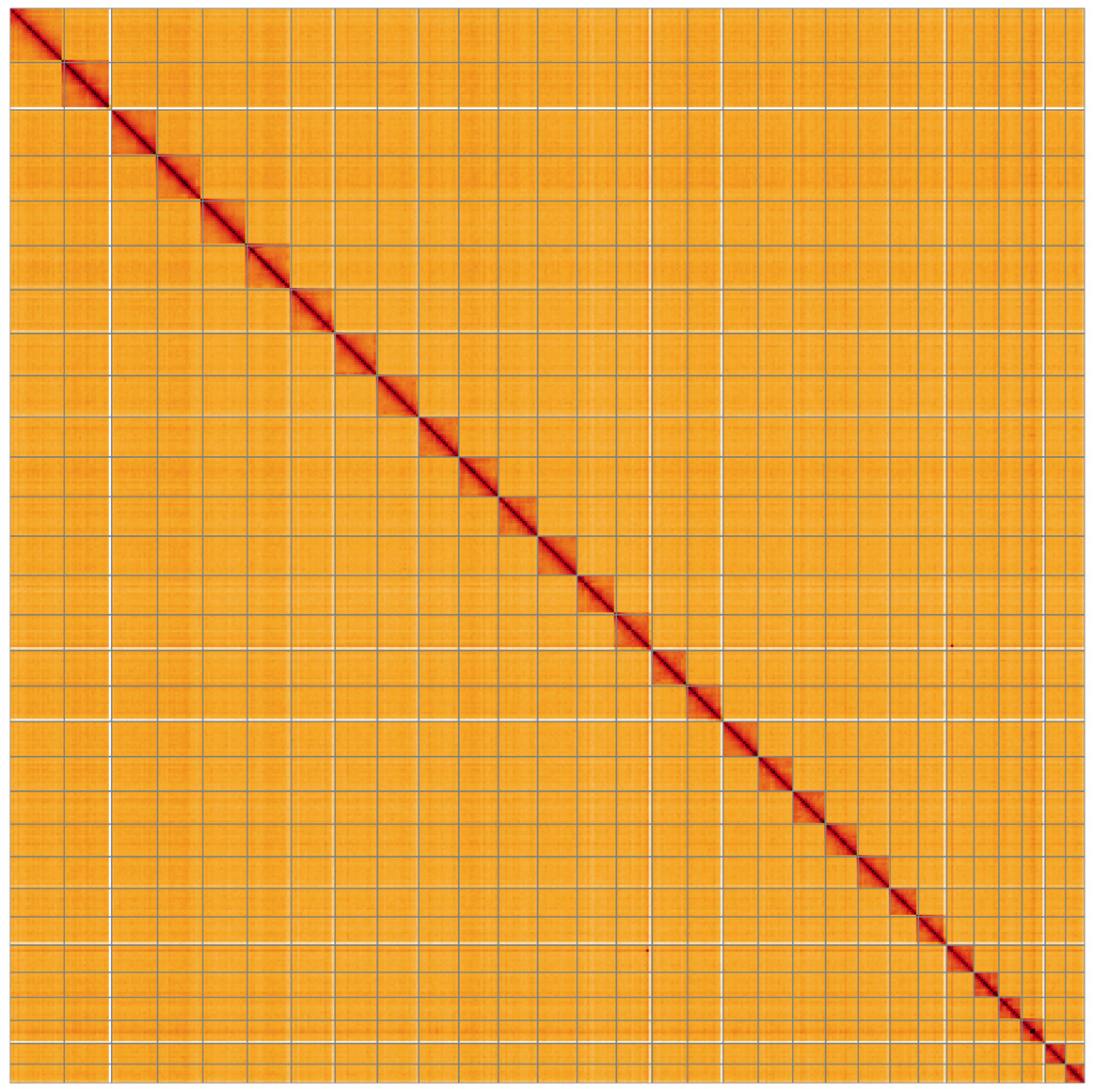
Genome assembly of *Achlya flavicornis*, ilAchFlav2.1: Hi-C contact map of the ilAchFlav2.1 assembly, visualised using HiGlass. Chromosomes are shown in order of size from left to right and top to bottom. An interactive version of this figure may be viewed at https://genome-note-higlass.tol.sanger.ac.uk/l/?d=IccbuLyRRCyTBelY_xuWEA.

**Table 2.** Chromosomal pseudomolecules in the genome assembly of *Achlya flavicornis*, ilAchFlav2.

INSDC accession	Chromosome	Length (Mb)	GC%
OX392467.1	1	22.52	37.0
OX392468.1	2	19.58	37.5
OX392469.1	3	19.03	37.0
OX392470.1	4	18.7	37.5
OX392472.1	5	18.19	37.5
OX392473.1	6	18.07	37.0
OX392474.1	7	17.49	37.0
OX392475.1	8	17.08	37.0
OX392476.1	9	16.53	37.0
OX392477.1	10	16.39	37.0
OX392478.1	11	16.28	37.0
OX392479.1	12	16.27	37.0
OX392480.1	13	16.25	36.5
OX392481.1	14	14.81	37.0
OX392482.1	15	14.67	37.0
OX392483.1	16	14.66	37.0
OX392484.1	17	14.53	37.5
OX392485.1	18	14.29	37.5
OX392486.1	19	13.6	37.5
OX392487.1	20	13.47	37.5
OX392488.1	21	13.09	37.0
OX392489.1	22	11.76	37.5
OX392490.1	23	11.73	37.5
OX392491.1	24	11.24	37.5
OX392492.1	25	10.32	37.5
OX392493.1	26	9.55	37.5
OX392494.1	27	9.54	39.5
OX392495.1	28	8.5	38.0
OX392496.1	29	7.94	38.0
OX392471.1	Z	18.45	37.0
OX392497.1	MT	0.02	19.0

The estimated Quality Value (QV) of the final assembly is 65.6 with *k*-mer completeness of 100%, and the assembly has a BUSCO v5.3.2 completeness of 98.8% (single = 98.5%, duplicated = 0.3%), using the lepidoptera_odb10 reference set ( *n* = 5,286).

Metadata for specimens, spectral estimates, sequencing runs, contaminants and pre-curation assembly statistics can be found at https://links.tol.sanger.ac.uk/species/753485.

## Genome annotation report

The *Achlya flavicornis* genome assembly (GCA_947623365.1) was annotated using the Ensembl rapid annotation pipeline ( Table 1; https://rapid.ensembl.org/Achlya_flavicornis_GCA_947623365.1/Info/Index). The resulting annotation includes 17,770 transcribed mRNAs from 17,588 protein-coding genes.

## Methods

### Sample acquisition and nucleic acid extraction

A male *Achlya flavicornis* (specimen ID Ox002041, individual ilAchFlav2) was collected from Wytham Woods, Oxfordshire, UK (latitude 51.77, longitude –1.34) on 2022-03-07, using a light trap. The specimen was collected and identified by Liam Crowley (University of Oxford) and preserved on dry ice.

DNA was extracted at the Tree of Life laboratory, Wellcome Sanger Institute (WSI). The ilAchFlav2 sample was weighed and dissected on dry ice with tissue set aside for Hi-C sequencing. Head and thorax tissue was disrupted using a Nippi Powermasher fitted with a BioMasher pestle. High molecular weight (HMW) DNA was extracted using the Qiagen MagAttract HMW DNA extraction kit. HMW DNA was sheared into an average fragment size of 12–20 kb in a Megaruptor 3 system with speed setting 30. Sheared DNA was purified by solid-phase reversible immobilisation using AMPure PB beads with a 1.8X ratio of beads to sample to remove the shorter fragments and concentrate the DNA sample. The concentration of the sheared and purified DNA was assessed using a Nanodrop spectrophotometer and Qubit Fluorometer and Qubit dsDNA High Sensitivity Assay kit. Fragment size distribution was evaluated by running the sample on the FemtoPulse system.

### Sequencing

Pacific Biosciences HiFi circular consensus DNA sequencing libraries were constructed according to the manufacturers’ instructions. DNA sequencing was performed by the Scientific Operations core at the WSI on a Pacific Biosciences SEQUEL II (HiFi) instrument. Hi-C data were also generated from remaining head and thorax tissue of ilAchFlav2 using the Arima2 kit and sequenced on the Illumina NovaSeq 6000 instrument.

### Genome assembly, curation and evaluation

Assembly was carried out with Hifiasm ( Cheng *et al.*, 2021) and haplotypic duplication was identified and removed with purge_dups ( Guan *et al.*, 2020). The assembly was scaffolded with Hi-C data ( Rao *et al.*, 2014) using YaHS ( Zhou *et al.*, 2023). The assembly was checked for contamination and corrected as described previously ( Howe *et al.*, 2021). Manual curation was performed using HiGlass ( Kerpedjiev *et al.*, 2018) and PretextView ( Harry, 2022). The mitochondrial genome was assembled using MitoHiFi ( Uliano-Silva *et al.*, 2023), which runs MitoFinder ( Allio *et al.*, 2020) or MITOS ( Bernt *et al.*, 2013) and uses these annotations to select the final mitochondrial contig and to ensure the general quality of the sequence.

A Hi-C map for the final assembly was produced using bwa-mem2 ( Vasimuddin *et al.*, 2019) in the Cooler file format ( Abdennur & Mirny, 2020). To assess the assembly metrics, the *k*-mer completeness and QV consensus quality values were calculated in Merqury ( Rhie *et al.*, 2020). This work was done using Nextflow ( Di Tommaso *et al.*, 2017) DSL2 pipelines “sanger-tol/readmapping” ( Surana *et al.*, 2023a) and “sanger-tol/genomenote” ( Surana *et al.*, 2023b). The genome was analysed within the BlobToolKit environment ( Challis *et al.*, 2020) and BUSCO scores ( Manni *et al.*, 2021; Simão *et al.*, 2015) were calculated.

Table 3 contains a list of relevant software tool versions and sources.

**Table 3.** Software tools: versions and sources.

Software tool	Version	Source
BlobToolKit	4.1.7	https://github.com/blobtoolkit/blobtoolkit
BUSCO	5.3.2	https://gitlab.com/ezlab/busco
Hifiasm	0.16.1-r375	https://github.com/chhylp123/hifiasm
HiGlass	1.11.6	https://github.com/higlass/higlass
Merqury	MerquryFK	https://github.com/thegenemyers/MERQURY.FK
MitoHiFi	2	https://github.com/marcelauliano/MitoHiFi
PretextView	0.2	https://github.com/wtsi-hpag/PretextView
purge_dups	1.2.3	https://github.com/dfguan/purge_dups
sanger-tol/genomenote	v1.0	https://github.com/sanger-tol/genomenote
sanger-tol/readmapping	1.1.0	https://github.com/sanger-tol/readmapping/tree/1.1.0
YaHS	yahs-1.1.91eebc2	https://github.com/c-zhou/yahs

### Genome annotation

The BRAKER2 pipeline ( Brůna *et al.*, 2021) was used in the default protein mode to generate annotation for the *Achlya flavicornis* assembly (GCA_947623365.1) in Ensembl Rapid Release.

### Wellcome Sanger Institute – Legal and Governance

The materials that have contributed to this genome note have been supplied by a Darwin Tree of Life Partner. The submission of materials by a Darwin Tree of Life Partner is subject to the **‘Darwin Tree of Life Project Sampling Code of Practice’**, which can be found in full on the Darwin Tree of Life website here. By agreeing with and signing up to the Sampling Code of Practice, the Darwin Tree of Life Partner agrees they will meet the legal and ethical requirements and standards set out within this document in respect of all samples acquired for, and supplied to, the Darwin Tree of Life Project.

Further, the Wellcome Sanger Institute employs a process whereby due diligence is carried out proportionate to the nature of the materials themselves, and the circumstances under which they have been/are to be collected and provided for use. The purpose of this is to address and mitigate any potential legal and/or ethical implications of receipt and use of the materials as part of the research project, and to ensure that in doing so we align with best practice wherever possible. The overarching areas of consideration are:

•     Ethical review of provenance and sourcing of the material

•     Legality of collection, transfer and use (national and international) 

Each transfer of samples is further undertaken according to a Research Collaboration Agreement or Material Transfer Agreement entered into by the Darwin Tree of Life Partner, Genome Research Limited (operating as the Wellcome Sanger Institute), and in some circumstances other Darwin Tree of Life collaborators.

## Data Availability

European Nucleotide Archive: *Achlya flavicornis* (yellow horned). Accession number PRJEB56908; https://identifiers.org/ena.embl/PRJEB56908. ( Wellcome Sanger Institute, 2022) The genome sequence is released openly for reuse. The *Achlya flavicornis* genome sequencing initiative is part of the Darwin Tree of Life (DToL) project. All raw sequence data and the assembly have been deposited in INSDC databases. Raw data and assembly accession identifiers are reported in Table 1.

## Author information

Members of the University of Oxford and Wytham Woods Genome Acquisition Lab are listed here: https://doi.org/10.5281/zenodo.4789928.

Members of the Darwin Tree of Life Barcoding collective are listed here: https://doi.org/10.5281/zenodo.4893703.

Members of the Wellcome Sanger Institute Tree of Life programme are listed here: https://doi.org/10.5281/zenodo.4783585.

Members of Wellcome Sanger Institute Scientific Operations: DNA Pipelines collective are listed here: https://doi.org/10.5281/zenodo.4790455.

Members of the Tree of Life Core Informatics collective are listed here: https://doi.org/10.5281/zenodo.5013541.

Members of the Darwin Tree of Life Consortium are listed here: https://doi.org/10.5281/zenodo.4783558.
