# Supplementary material for: The genome sequence of the Yellow Horned moth, Achlya flavicornis (Linnaeus, 1758)
Source: Wellcome Open Res. 2025 Feb 19;8:458. Originally published 2023 Oct 12. [Version 2] doi: 10.12688/wellcomeopenres.19846.2 (PMC11851003; doi:10.12688/wellcomeopenres.19846.2)
Supplement: Peer review history

# Reviewer report: Reviewer response for version 2

**DOI:** 10.21956/wellcomeopenres.26274.r119257

**Published:** 2025-02-24

**Authors:** Kay Lucek

**Affiliations:** 1 Biodiversity Genomics Laboratory, University of Neuchâtel, Neuchâtel, Switzerland

The authors present the chromosome level genome assembly of a male specimen of the Yellow Horned moth *Achlya flavicornis*. The assembly consists of 30 chromosomes including the Z chromosome. The assembly is highly complete as revealed by the high BUSCO score but not fully phased. Sequencing and genome assembly follow the current state of the art and use established methods.

 Overall, the presented assembly will be of great value to study genome architecture as well as genome size evolution in Lepidoptera. Moreover the reference genome provides the opportunity to study the existing subspecies in the UK and across the specie’s range.

Are sufficient details of methods and materials provided to allow replication by others?

Yes

Is the rationale for creating the dataset(s) clearly described?

Yes

Are the datasets clearly presented in a useable and accessible format?

Yes

Are the protocols appropriate and is the work technically sound?

Yes

Reviewer Expertise:

Biodiversity genomics

I confirm that I have read this submission and believe that I have an appropriate level of expertise to confirm that it is of an acceptable scientific standard.

# Reviewer report: Reviewer response for version 1

**DOI:** 10.21956/wellcomeopenres.21979.r81337

**Published:** 2024-05-23

**Authors:** Laurence Després

**Affiliations:** 1 Universite Grenoble Alpes, Universite Savoie Mont Blanc, Saint-Martin-d'Hères, Auvergne-Rhône-Alpes, France

The authors report on the  chromosome-level genome assembly of the Yellow Horned, *Achlya flavicornis,* a common western Palaearctic Drepanid moth that appears to be increasing in the last decades in UK. The genome assembly is of good quality and was constructed using appropriate methods : Pac Bio HiFi long reads (49-fold coverage) and Hi-C data to confirm scaffolding, manual curation of the final assembly, and following the high standard of the Darwin Tree of Life Project for results presentation.

 The final assembly has a total length of 444.6 Mb and includes 30 chromosomes: 29 autosomes and the sexual Z chromosome that was identified based on its synteny with another Drepanid moth, *Habrosyne pyritoides* (the sequenced specimen is a male). The mitogenome was also assembled. The quality of the assembly was further assessed by the proportion of complete BUSCO genes recovered, which is very high (>98% of Lepidoptera database BUSCO genes). Automated genome annotation was performed using  the BREAKER2 pipeline and 17,588 protein-coding genes were predicted.

Are sufficient details of methods and materials provided to allow replication by others?

Yes

Is the rationale for creating the dataset(s) clearly described?

Yes

Are the datasets clearly presented in a useable and accessible format?

Yes

Are the protocols appropriate and is the work technically sound?

Yes

Reviewer Expertise:

evolutionary ecology and population genomics of alpine butterflies; phylogenomics, demographic inferences, adaptation, elevational gradient.

I confirm that I have read this submission and believe that I have an appropriate level of expertise to confirm that it is of an acceptable scientific standard.

# Reviewer report: Reviewer response for version 1

**DOI:** 10.21956/wellcomeopenres.21979.r79613

**Published:** 2024-05-10

**Authors:** Fahad Alqahtani

**Affiliations:** 1 King Abdulaziz City for Science and Technology, Riyadh, Saudi Arabia

In "The genome sequence of the Yellow Horned, *Achlya flavicornis* (Linnaeus, 1758)," the authors have achieved a highly complete genome assembly at the chromosome level for a male yellow horned, *Achlya flavicornis* (Linnaeus, 1758).

 The genome assembly was reconstructed using two sequencing technologies: Pacific Biosciences SEQUEL II and Hi-C Illumina. The completeness of the genome assembly was assessed using BUSCO analysis, which indicated a genome size of 444.6 Mb bp, with 98.8% of common genes being completely present.

 There are a few minor comments that should be addressed:

- In Table 1, the authors report the number of contigs as 64, while NCBI lists 63.
- "Pretext" in the Genome Assembly section should be corrected to "PretextView" to match the name in Table 3.

Are sufficient details of methods and materials provided to allow replication by others?

Yes

Is the rationale for creating the dataset(s) clearly described?

Yes

Are the datasets clearly presented in a useable and accessible format?

Yes

Are the protocols appropriate and is the work technically sound?

Yes

Reviewer Expertise:

Bioinformatics

I confirm that I have read this submission and believe that I have an appropriate level of expertise to confirm that it is of an acceptable scientific standard, however I have significant reservations, as outlined above.
